# Comparative Study of Levels of Serum Bilirubin, Serum Transaminase, Serum Alkaline Phosphatase, and Prothrombin Time After Laparoscopic Cholecystectomy and Open Cholecystectomy

**DOI:** 10.7759/cureus.60296

**Published:** 2024-05-14

**Authors:** Rahul Sarma, Sushmita Ray, Nirupam Konwar Baishya, Wahida Sultana

**Affiliations:** 1 General Surgery, GNRC Hospital, Guwahati, IND; 2 General Surgery, Fakhruddin Ali Ahmed Medical College and Hospital, Barpeta, IND; 3 Radiodiagnosis, Fakhruddin Ali Ahmed Medical College and Hospital, Barpeta, IND

**Keywords:** prothrombin time, serum alkaline phosphatase, serum transaminase, serum bilirubin, open cholecystectomy, laparoscopic cholecystectomy

## Abstract

Laparoscopic cholecystectomy (LC) is universally accepted as the gold standard treatment for symptomatic gallstones. However, it has some drawbacks. Some of the major drawbacks of LC include increased bile duct injuries and longer operation time. Furthermore, it may cause changes in the body systems, such as alterations in acid-base, pulmonary status, cardiovascular system, and liver function. Thus far, no causes for these changes have been identified. This study aimed to evaluate the effect of laparoscopic and open cholecystectomy on liver enzymes, prothrombin time (PT), and serum bilirubin. In the current study, we found significant increases in aspartate transferase (AST), alanine transaminase (ALT), and total bilirubin, on day 1 and day 3 after LC but no significant change in alkaline phosphatase (ALKP) and PT. It is important for surgeons to know about these transient changes in the immediate postoperative period to avoid misdiagnosis and adopt proper treatment and management.

## Introduction

Laparoscopic cholecystectomy (LC) is universally accepted as the gold standard treatment for symptomatic gallstones. Because LC is associated with reduced pain and shorter hospital stays and, most importantly, enables an early return to family, normal employment, and exercise, patient acceptance, preference, and demand have all increased dramatically [1]. It also has an excellent cosmetic outcome and a low risk of wound infection, hernia, nerve entrapment, and postoperative adhesions [1-4]. The main advantage of laparoscopic surgery is decreased tissue trauma [3-6].

However, LC has some drawbacks. Some of the major drawbacks of LC include increased bile duct injuries and longer operation time [1,2]. Furthermore, it may result in modifications to the acid-base, pulmonary state, cardiovascular system, and liver function, among other bodily systems [1,2]. Thus far, no causes for these changes have been identified. These alterations may be related to hepatocellular dysfunction brought on by diathermy, carbon dioxide-induced pneumoperitoneum, hepatic artery injury, or side effects of general anesthesia [7-11]. A disruption in blood flow to the liver can not only cause liver dysfunction resulting in increased levels of liver enzymes like aspartate transaminases, alanine transaminase, and alkaline phosphatase (ALKP), but it can also disrupt protein production in hepatocytes, including coagulation factors, which may lead to increased prothrombin time (PT) [12-14]. If any changes in liver enzymes occur after surgery, it can lead to a challenge in the management of the patient in the postoperative period. A more extensive assessment of the postoperative alterations in liver enzymes after LC has not been conducted. In order to assess and determine the changes in liver function tests following open and LC, the current study was designed.

## Materials and methods

This is a prospective observational study conducted on patients admitted to Fakhruddin Ali Ahmed Medical College & Hospital, a tertiary care hospital in North East India, for a period of one year from July 18, 2021, to July 17, 2022. A pre-structural proforma was used to collect baseline data and detailed clinical history with clinical examination, and relevant investigations were done on participating individuals. The sampling design was purposive with a sample size of 100 patients (50 open cholecystectomy and 50 LP). The patients who fulfilled the selection criteria were explained about the nature of the study, and written informed consent was obtained. The inclusion criteria were as follows: 1) cholelithiasis patients undergoing cholecystectomy (open or lap), 2) age group of 18-60 years, 3) patients who had preoperatively normal liver function tests, and 4) patients who gave written consent for the procedure. The exclusion criteria were as follows: 1) patients who had undergone previous abdominal surgery; 2) patients having gallstone-related complications; 3) presence of cardiac or hepatic disease; 4) bile duct-related injury, obstruction, or infection; 5) choledocholithiasis; 6) pregnant females; 7) cases of pancreatitis; 8) bleeding or clotting disorder; 9) cases in which malignancy was suspected; 10) comorbidities like diabetes mellitus, chronic liver diseases, chronic renal diseases, cardiac disease, and hepatitis B or C virus infections; and 11) patients who had undergone an endoscopic retrograde cholangiopancreatography (ERCP) procedure in the last 10 days.

The Institutional Ethics Committee of Fakhruddin Ali Ahmed Medical College & Hospital issued approval (ref no. FAAMC&H/IEC_PG/498/2020/6885).

Study procedures

**Figure 1:**
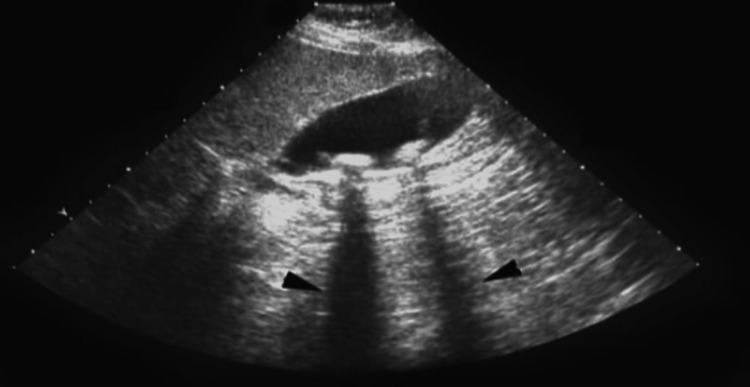
Ultrasound of the gallbladder. Arrows indicate posterior acoustic shadowing from gallstones.

Individuals who consented to take part in the research underwent standard laboratory and clinical testing, including ultrasonography (USG) (Figure 1). Liver function tests, such as total bilirubin, alanine transaminase (ALT), aspartate transaminase (AST), ALKP, and PT, were performed as part of the preoperative studies.

In our facility, the anesthetic regimen was the same for both types of cholecystectomies. Following surgery, the same intravenous antibiotic infusion treatment was administered to each patient. Drugs that are hepatotoxic and nephrotoxic were not used.

AST, ALT, ALKP, PT, and serum bilirubin were assessed in all the patients chosen for the study preoperatively once and then postoperatively on day 1, day 3, and day 7.

## Results

**Figure 2:**
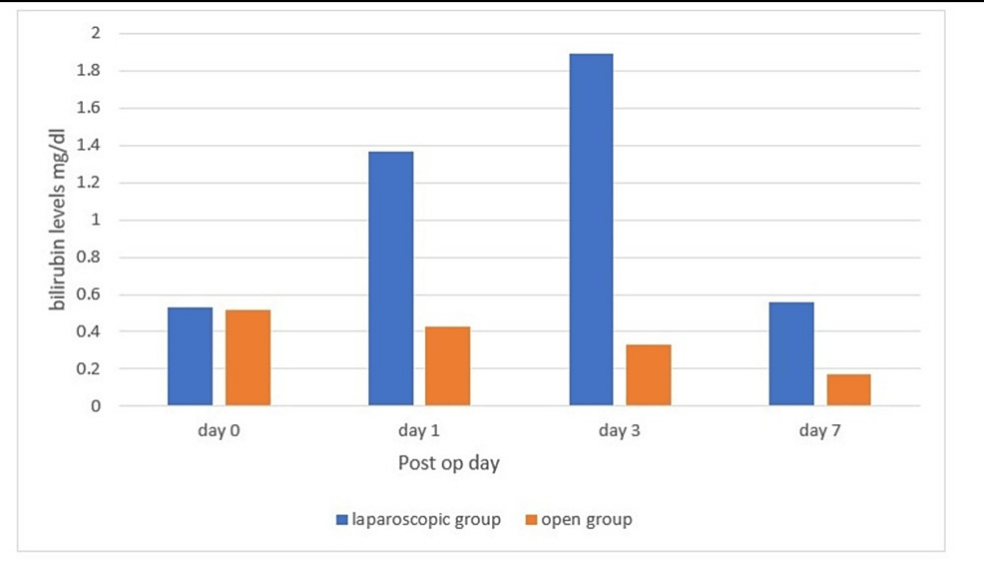
Bilirubin distribution at day 0, day 1, day 3, and day 7 (p < 0.05)

In the study, after analyzing bilirubin distribution, it was found that in the laparoscopic group, the preoperative serum total bilirubin was 0.53 ± 0.15 mg/dl, and in the open group, it was 0.52 ± 0.28 mg/dL (p > 0.05). In the laparoscopic group, the postoperative serum total bilirubin on day 1 was 1.37 ± 0.64 mg/dL, and in the open group, it was 0.43 ± 0.26 mg/dL (p < 0.05). In the laparoscopic group, the postoperative serum total bilirubin on day 3 was 1.89 ± 0.56 mg/dL, and in the open group, it was 0.77 ± 0.33 mg/dL (p < 0.05). In the laparoscopic group, the postoperative serum total bilirubin on day 7 was 0.56 ± 0.20 mg/dL, and in the open group, it was 0.53 ± 0.17 mg/dL (p>0.05). The above findings are represented in Figure 2.

**Figure 3:**
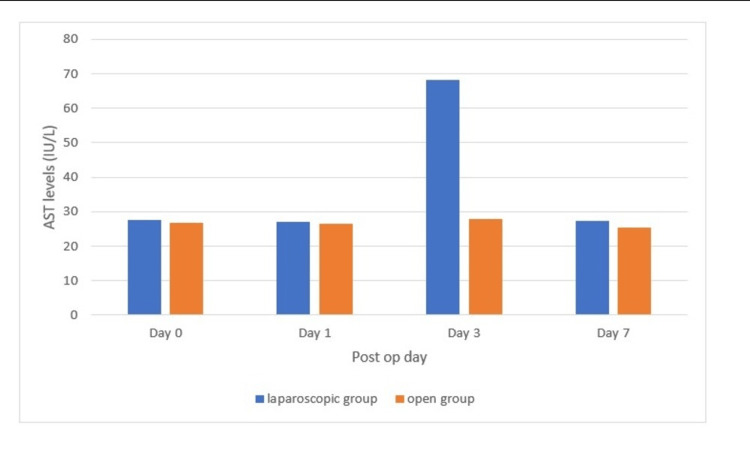
Aspartate aminotransferase distribution at day 0, day 1, day 3, and day 7 (p < 0.05)

Analyzing the AST distribution, it was found that in the laparoscopic group, the preoperative AST was 27.46 ± 6.38 IU/L, and in the open group, it was 26.89 ± 5.76 IU/L (p > 0.05). In the laparoscopic group, the postoperative AST on day 1 was 26.94 ± 6.37 IU/L, and in the open group, it was 26.56 ± 5.69 IU/L (p < 0.05). In the laparoscopic group, the postoperative AST on day 3 was 68.31 ± 7.61 IU/L, and in the open group, it was 27.77 ± 3.71 IU/L (p < 0.05). In the laparoscopic group, the postoperative AST on day 7 was 27.42 ± 5.46 IU/L, and in the open group, it was 25.45 ± 4.03 IU/L (p > 0.05). The above findings are represented in Figure 3.

**Figure 4:**
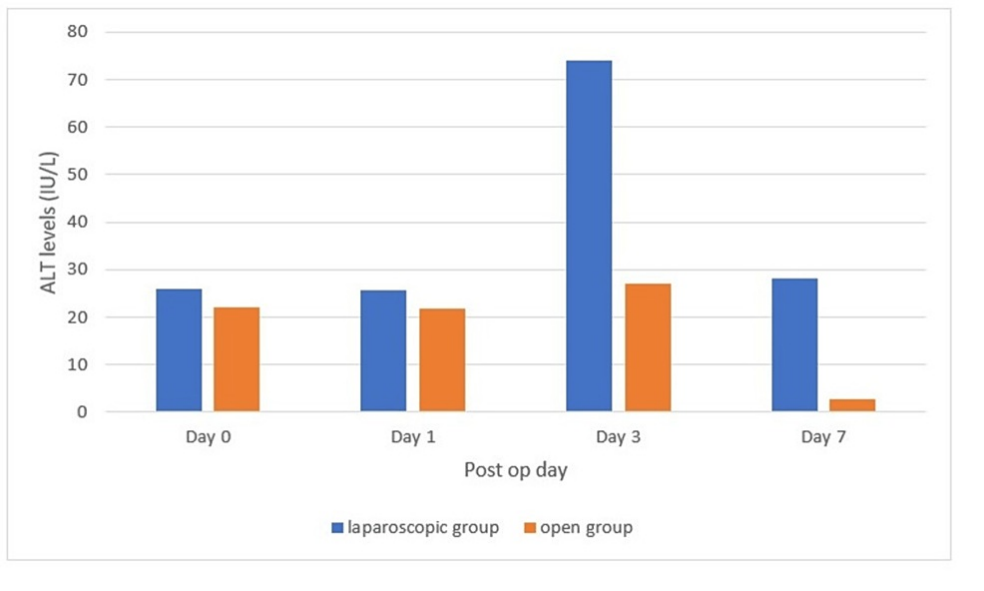
Alanine transaminase distribution at day 0, day 1, day 3, and day 7 (p < 0.05)

Analyzing the ALT distribution, it was found that in the laparoscopic group, the preoperative ALT was 26.06 ± 7.06 IU/L, and in the open group, it was 22.01 ± 6.32 IU/L (p > 0.05). In the laparoscopic group, the postoperative ALT on day 1 was 25.56 ± 7.04 IU/L, and in the open group, it was 21.67 ± 6.27 IU/L (p < 0.05). In the laparoscopic group, the postoperative ALT on day 3 was 73.94 ± 11.20 IU/L, and in the open group, it was 26.96 ± 8.44 IU/L (p < 0.05). In the laparoscopic group, the postoperative ALT on day 7 was 28.22 ± 7.38 IU/L, and in the open group, it was 22.69 ± 5.67 IU/L (p > 0.05). The above findings are represented in Figure 4.

**Figure 5:**
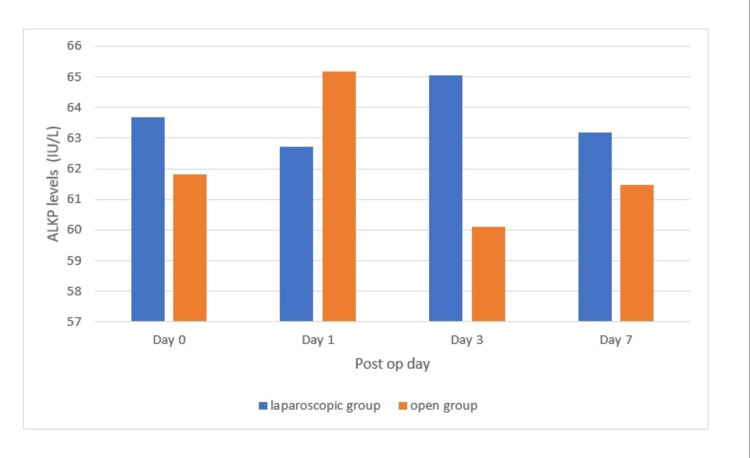
Alkaline phosphatase distribution at day 0, day 1, day 3, and day 7 (p > 0.05)

Analyzing the ALKP distribution, it was found that in the laparoscopic group, the preoperative ALKP was 63.69 ± 10.77 IU/L, and in the open group, it was 61.81 ± 10.59 IU/L (p > 0.05). In the laparoscopic group, the postoperative ALKP on day 1 was 62.73 ± 6.31 IU/L, and in the open group, it was 65.16 ± 7.26 IU/L (p > 0.05). In the laparoscopic group, the postoperative ALKP on day 3 was 65.05 ± 9.01 IU/L, and in the open group, it was 60.10 ± 7.21 IU/L (p > 0.05). In the laparoscopic group, the postoperative ALKP on day 7 was 63.17 ± 10.79 IU/L, and in the open group, it was 61.47 ± 10.67 IU/L (p > 0.05). The above findings are represented in Figure 5.

**Figure 6:**
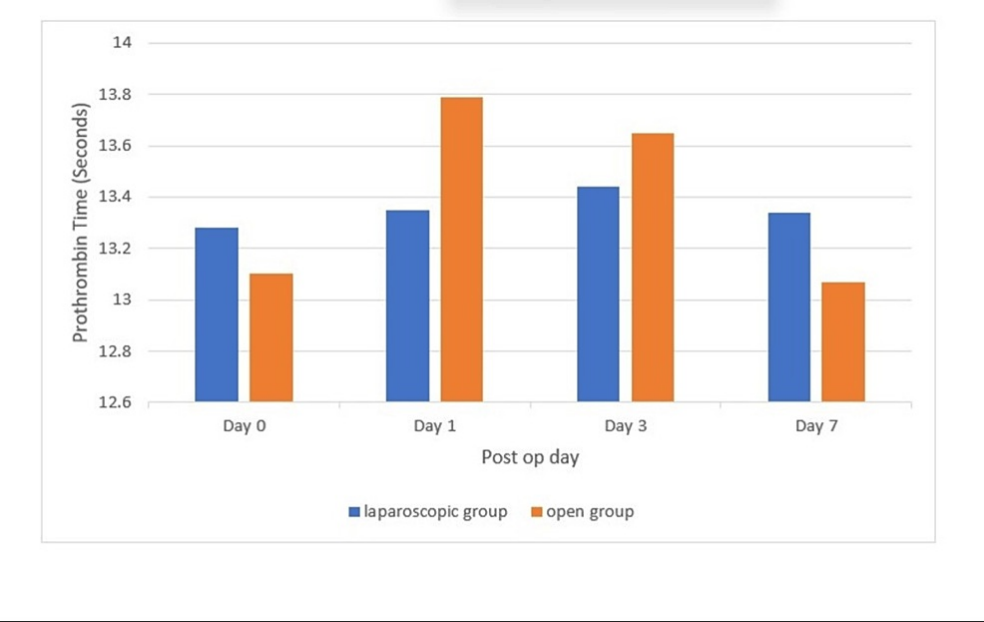
Prothrombin time distribution at day 0, day 1, day 3, and day 7 (p > 0.05)

Analyzing the PT distribution, it was found that in the laparoscopic group, the preoperative PT was 13.28 ± 1.05 seconds, and in the open group, it was 13.10 ± 1.01 seconds (p > 0.05). In the laparoscopic group, the postoperative PT on day 1 was 13.35 ± 1.33 seconds, and in the open group, it was 13.79 ± 1.07 seconds (p > 0.05). In the laparoscopic group, the postoperative PT on day 3 was 13.44 ± 1.13 seconds, and in the open group, it was 13.65 ± 1.15 seconds (p > 0.05). In the laparoscopic group, the postoperative PT on day 7 was 13.34 ± 1.11 seconds, and in the open group, it was 13.07 ± 1.28 seconds (p > 0.05). The above findings are represented in Figure 6.

## Discussion

This study aimed to compare the effects of open cholecystectomy and LC on serum bilirubin, PT, and liver enzymes (serum transaminase and ALKP). This study included the distribution of age, gender, duration of surgery, AST, ALT, ALKP, PT, and serum bilirubin among the two groups. Several facts have been enumerated, some of which are per the literature while a few differ. In the current study, we found significant increases in AST, ALT, and total bilirubin on day 1 and day 3 after LC.

Guven et al. studied liver enzymes and LDH post-open and LC [15]. They discovered that after LC, there was a considerable increase in AST, ALT, and LDH but not ALP [15]. Except for ALP, our study also showed comparable results for AST and ALT.

According to Hasukic et al.'s research, individuals receiving LC had significantly higher levels of AST and ALT 48 hours after surgery compared to those undergoing open surgery [16]. They did note, however, that after 48 hours, LDH and ALP did not change [16]. Similar rises in AST and ALT were also observed in our investigation.

In a related study, Halevy et al. reported increases in AST, ALT, ALP, and total bilirubin levels of 73%, 82%, 53%, and 14%, respectively, following LC [17]. In keeping with their study, ours also showed comparable increases in AST, ALT, ALP, and total bilirubin. The first study on changes in liver enzymes following LC was conducted in 1994 by Halevy et al. [17]. These effects might be brought on by elevated intraperitoneal pressure or by the gallbladder retracting and compressing the liver [17]. The effects of anesthesia and the introduction of surgical diathermy to the liver bed are two other potential explanations. However, since the anesthetic protocol was the same for the laparoscopic and open groups, it is not possible to attribute the differences in the laparoscopic surgery to the effects of anesthetic medication alone [17]. The pneumoperitoneum brought on by carbon dioxide gas can be the primary cause. Changes in liver enzymes are most likely caused by CO_2_ pneumoperitoneum. To perform an LC, the pneumoperitoneum must be created. Reduced blood flow is caused by the formation of pneumoperitoneum, which impairs tubular and glomerular filtration and results in aberrant biochemical values.

The rise in AST, ALT, and bilirubin was significant statistically but non-significant in the case of the ALP study done by Bellad and Sahu, which showed that there was a significant increase in bilirubin (total), AST, ALT, and ALP from baseline and decrease of serum albumin and total proteins in patients who underwent LC [18]. The study done by them also supported our results.

The PT of patients was examined in the study for both the LC and open cholecystectomy groups, and it was discovered that not every patient had the same outcome. The results are consistent with a study done by Garg et al., who likewise found no difference in PT [19].

Omari and Bani-Hani studied the serum levels of eight parameters of liver function tests preoperatively and 24 hours [20] after surgery in 142 patients who underwent LC, 23 patients who underwent open cholecystectomy, and 25 patients who underwent a conventional hernia repair [20]. Twelve mmHg of carbon dioxide intra-abdominal pressure was maintained. It appeared that the pneumoperitoneum played a major role in these elevations of parameters [20]. They found that 83% of the patients showed more than a 100% increase in at least one parameter, 43% showed an increase in two or more parameters, and 23% showed an increase in three or more parameters [20].

The limitation of the present study is that it could not be conducted on the patients suffering from hepatic disease, and hence the trend of change of liver enzyme levels in such patients could not be evaluated, which would have otherwise contributed more to its significance.

## Conclusions

Our study has demonstrated that LC produces significant changes in serum transaminase and serum bilirubin in the immediate postoperative period. However, these changes spontaneously reverted to preoperative levels over a week resulting in no morbidity or mortality. Nevertheless, surgeons need to know about these transient changes in the immediate postoperative period to prevent any untoward interventions in the perioperative period and to practice caution while considering other differential diagnoses. Overall, this can prompt better and more targeted care for cholecystectomy patients.

## References

[1] Wellwood J, Sculpher MJ, Stoker D (1998). Randomised controlled trial of laparoscopic versus open mesh repair for inguinal hernia: outcome and cost. BMJ.

[2] Zacks SL, Sandler RS, Rutledge R, Brown RS Jr (2002). A population-based cohort study comparing laparoscopic cholecystectomy and open cholecystectomy. Am J Gastroenterol.

[3] Novitsky YW, Litwin DE, Callery MP (2004). The net immunologic advantage of laparoscopic surgery. Surg Endosc.

[4] Buunen M, Gholghesaei M, Veldkamp R, Meijer DW, Bonjer HJ, Bouvy ND (2004). Stress response to laparoscopic surgery: a review. Surg Endosc.

[5] Corcione F, Esposito C, Cuccurullo D (2005). Advantages and limits of robot-assisted laparoscopic surgery: preliminary experience. Surg Endosc.

[6] Shih YC, Sheau-Farn Max Liang Bridging research and good practices towards patients welfare. Proceedings of the 4th International Conference on Healthcare Ergonomics and Patient Safety (HEPS), Taipei, Taiwan.

[7] Glantzounis GK, Tselepis AD, Tambaki AP (2001). Laparoscopic surgery-induced changes in oxidative stress markers in human plasma. Surg Endosc.

[8] Eryılmaz HB, Memiş D, Sezer A, Inal MT (2012). The effects of different insufflation pressures on liver functions assessed with LiMON on patients undergoing laparoscopic cholecystectomy. ScientificWorldJournal.

[9] Gupta R, Kaman L, Dahiya D, Gupta N, Singh R (2013). Effects of varying intraperitoneal pressure on liver function tests during laparoscopic cholecystectomy. J Laparoendosc Adv Surg Tech A.

[10] Ahmad NZ (2011). Routine testing of liver function before and after elective laparoscopic cholecystectomy: is it necessary?. JSLS.

[11] Singal R, Singal RP, Sandhu K, Singh B, Bhatia G, Khatri A, Sharma BP (2015). Evaluation and comparison of postoperative levels of serum bilirubin, serum transaminases and alkaline phosphatase in laparoscopic cholecystectomy versus open cholecystectomy. J Gastrointest Oncol.

[12] Koirala R, Shakya VC, Khania S, Adhikary S, Agrawal CS (2012). Rise in liver enzymes after laproscopic cholecystectomy: a transient phenomenon. Nepal Med Coll J.

[13] Khalaf R, Al-Luwaizi S, Hamad A, Coll Coll, Al-Luwaizi K, Hamad S (2013). Jumhori Teaching Hospital. Ann Coll Med Mosul.

[14] Avraamidou A, Marinis A, Asonitis S, Perrea D, Polymeneas G, Voros D, Argyra E (2012). The impact of ischemic preconditioning on hemodynamic, biochemical and inflammatory alterations induced by intra-abdominal hypertension: an experimental study in a porcine model. Langenbecks Arch Surg.

[15] Guven HE, Oral S (2007). Liver enzyme alterations after laparoscopic cholecystectomy. J Gastrointestin Liver Dis.

[16] Hasukic S (2014). Co2-pneumoperitoneum in laparoscopic surgery: pathophysiologic effects and clinical significance. World J Laparosc Surg.

[17] Halevy A, Gold-Deutch R, Negri M (1994). Are elevated liver enzymes and bilirubin levels significant after laparoscopic cholecystectomy in the absence of bile duct injury?. Ann Surg.

[18] Bellad A, Sahu K (2019). An observational study on effect of carbon dioxide pneumoperitoneum on liver function test in laparoscopic cholecystectomy. Int Surg J.

[19] Garg PK, Teckchandani N, Hadke NS, Chander J, Nigam S, Puri SK (2009). Alteration in coagulation profile and incidence of DVT in laparoscopic cholecystectomy. Int J Surg.

[20] Omari A, Bani-Hani KE (2007). Effect of carbon dioxide pneumoperitoneum on liver function following laparoscopic cholecystectomy. J Laparoendosc Adv Surg Tech A.

